# Construction and Multicenter Diagnostic Verification of Intelligent Recognition System for Endoscopic Images From Early Gastric Cancer Based on YOLO-V3 Algorithm

**DOI:** 10.3389/fonc.2022.815951

**Published:** 2022-01-25

**Authors:** Zhendong Yao, Tao Jin, Boneng Mao, Bo Lu, Yefei Zhang, Sisi Li, Weichang Chen

**Affiliations:** ^1^ Department of Gastroenterology, The First Affiliated Hospital of Soochow University, Suzhou, China; ^2^ Department of Gastroenterology, Yixing People’s Hospital, Yixing, China; ^3^ Microsoft Teams Calling Meeting Device of Sharepoint Onedrive eXperience (Teams CMD SOX), Microsoft Ltd Co., Suzhou, China; ^4^ Department of Gastroenterology, The Second Affiliated Hospital of Soochow University, Suzhou, China; ^5^ Department of Gastroenterology, Civil Aviation Hospital of Shanghai, Shanghai, China

**Keywords:** Early gastric cancer, YOLO, endoscopy, Convolutional neural network, artificial intelligence

## Abstract

**Introduction:**

Endoscopy is an important tool for the diagnosis of early gastric cancer. Therefore, a combination of artificial intelligence and endoscopy has the ability to increase the speed and efficiency of early gastric cancer diagnosis. YOU ONLY LOOK ONCE (YOLO) is an advanced object detection depth neural network algorithm that has not been widely used in gastrointestinal image recognition.

**Objective:**

We developed an artificial intelligence system herein referred to as “EGC-YOLO” for the rapid and accurate diagnosis of endoscopic images from early gastric cancer.

**Methods:**

More than 40000 gastroscopic images from 1653 patients in Yixing people’s Hospital were used as the training set for the system, while endoscopic images from the other two hospitals were used as external validation test sets. The sensitivity, specificity, positive predictive value, Youden index and ROC curve were analyzed to evaluate detection efficiencies for EGC-YOLO.

**Results:**

EGC-YOLO was able to diagnose early gastric cancer in the two test sets with a high superiority and efficiency. The accuracy, sensitivity, specificity and positive predictive value for Test Sets 1 and 2 were 85.15% and 86.02%, 85.36% and 83.02%, 84.41% and 92.21%, and 95.22% and 95.65%, respectively. In Test Sets 1 and 2, the corresponding Threshold-values were 0.02, 0.16 and 0.17 at the maximum of the Youden index. An increase in Threshold-values was associated with a downward trend in sensitivity and accuracy, while specificity remained relatively stable at more than 80%.

**Conclusions:**

The EGC-YOLO system is superior for the efficient, accurate and rapid detection of early gastric cancer lesions. For different data sets, it is important to select the appropriate threshold-value in advance to achieve the best performance of the EGC-YOLO system.

## Introduction

Gastric cancer is one of the most common malignant tumors in the world, and the third leading cause of cancer-related death. Nearly 1 million new cases and 783000 gastric cancer-related deaths are reported each year (1, 2). The prognosis of patients with gastric cancer depends on the stage of cancer, with patients with advanced gastric cancer having poor prognosis. On the other hand, the 5-year survival rate of patients with early gastric cancer is more than 90%. This is because patients at the early stage can be treated directly through endoscopic mucosal resection (EMR) or endoscopic submucosal dissection (ESD). The operation is simple and hardly impacts the quality of life of the patients (3–5). Therefore, endoscopy is the standard method for early screening of gastric cancer. However judging between benign and malignant tissue under gastroscopy mainly depends on the diagnostic expertise of endoscopic physicians, and inexperienced endoscopic doctors often misdiagnose patients. Atrophic gastritis is a precancerous state that gives rise to more than 95% of gastric adenocarcinomas (6–8). The morphological features of early gastric cancer are difficult to distinguish from atrophic gastritis under white light endoscopy. Therefore, endoscopic physicians need long-term specialized training and a wealth of experience to correctly detect gastric cancer. Differences in expertise among endoscopic physicians are responsible for the unequal detection rates of early gastric cancer in different regions and different levels of hospitals. As a result, improving the efficiency of endoscopic diagnosis of early gastric cancer is the most effective measure to reduce the mortality rates associated with gastric cancer. Several endoscopic aids such as magnifying gastroscopy, chromoendoscopy and narrow band imaging (NBI) have been developed to improve the detection rate of early gastric cancer (9–14). Recently, there has been great progress in image recognition technology based on artificial intelligence (AI) and machine learning, which has been increasingly used in image recognition and auxiliary diagnosis in many fields of medicine. These areas include the identification or classification of skin cancer (15–17), radiation oncology (18–20), the diagnosis of retinopathy (21–23), histological classification of pathological biopsies (24–27), and the characterization of colorectal lesions.

Several multicenter studies using CNN to train and recognize images of early gastric cancer have been conducted with satisfactory results. However, in this study, we used the brand-new YOLOv3 algorithm for training and testing. The algorithm is a region-based convolution neural network characterized by high speed, strong versatility and low background error detection rate. We developed the EGC-YOLO diagnostic system based on artificial intelligence by training more than 40000 gastroscopic images to distinguish between early gastric cancer and benign lesion. We then used endoscopic images obtained from other databases to verify the performance of the diagnostic system, and obtained good results. Our findings indicated that EGC-YOLO has good potential for application in the intelligent diagnosis of early gastric cancer.

## Materials and Methods

### Equipment and Software Used for the Study

Convolutional Neural Network(CNN) related development software, PYTHON programming language, LINUX system, GPU: NVIDIA RTX 2080TI+NVIDIA GTX 1080TI.

### Data Collection and Grouping

The training set consisted of early gastric cancer cases with endoscopic images along with pathological examination confirming the diagnosis (endoscopic diagnosis of early gastric cancer, pathological type of moderate + anisotropic hyperplasia, severe anisotropic hyperplasia, intra-mucosal carcinoma) treated at the Yixing People’s Hospital from 2019 to 2020. The control group comprised of normal gastroscopic images taken by the same endoscopic machine during the same period (with pathological biopsy report supporting the endoscopic diagnosis), and non-gastric cancer cases that could be easily confused with early gastric cancer images, including (chronic superficial gastritis, chronic atrophic gastritis, warty gastritis, gastric ulcer, acute gastritis, erosive gastritis). A total of 1653 cases, involving 42,200 images of non-gastric cancer and 945 endoscopic images of early gastric cancer were included in the training set.

The test set comprised of 280 early gastric cancer images and 77 non-gastric cancer images from the Second Affiliated Hospital of Soochow University, and 159 early gastric cancer images and 77 non-gastric cancer images from Civil Aviation Hospital of Shanghai.

### Construction of the EGC-YOLO Algorithm

We used the general architecture of YOLOv3, with DarkNet53 as the backbone network and a three-layer spatial pyramid as neck to increase detection accuracy. In the detection head, BCE Loss was used as the target loss function, and a branch and loss function specifically optimized for IoU was added to the original YOLOv3. For the detection of early cancer, classification accuracy was more important than the detection area, so we set a larger weight for classification loss.

We used the Xavier initialization method to randomly generate the network parameters so that the activation function inputs for each layer at the beginning of the training phase were in a reasonable interval to ensure convergence speed.

Since our training data set was not as large as data in the YOLOv3 network, training directly using the parameters generated by the Xavier initialization method could have resulted in over fitting. Consequently, we first pre-trained on the image classification task of Image Net and the object detection task of the COCO dataset to obtain the parameters of the DarkNet53 backbone network. We then added a three-layer pyramid detection neck and fine-tuned on the early cancer data set. In order to match the training parameters with the early cancer data set as closely as possible, we homogenized the images from the Image Net and COCO datasets by using the mean and variance of the early cancer training set.

In the fine tuning phase of the network, we used 640x640 images as input and 64 images as a batch for one round of iteration. Due to GPU memory limitation, a batch was divided into 32 divisions. A total of 100 epochs were done, with the first 2 epochs using a cosine learning rate of 0.01 as the warm-up training, and the learning rate becoming 0.001 after the warm-up.

**Figure 1:**
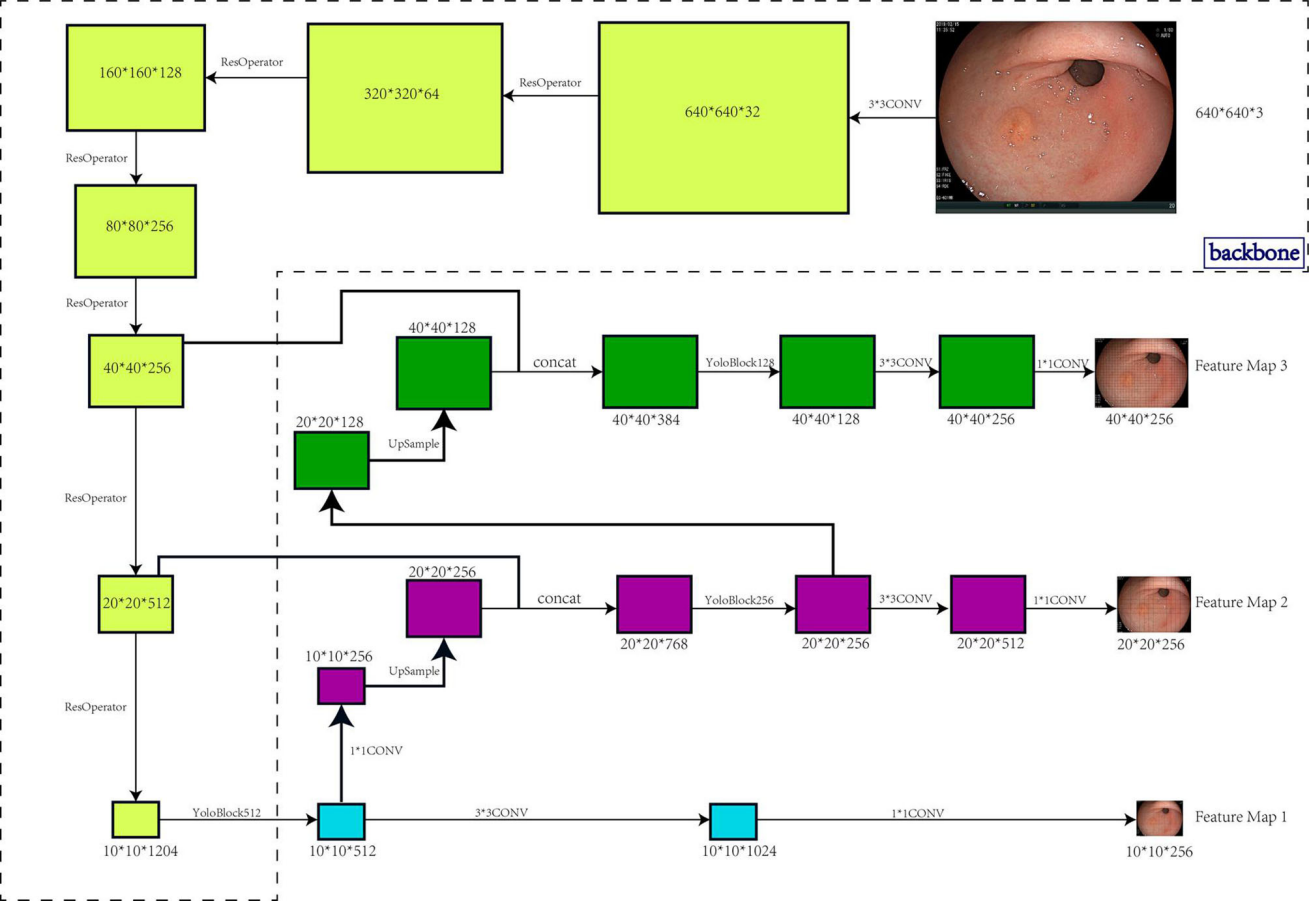
Architecture and workflow of the EGC-YOLO system. The target detection model was divided into three parts, the backbone, the bottleneck network, and the detection head. The backbone network was responsible for the feature extraction of the image. 640x640x3 image raw input was transformed into 40x40x256 feature maps using 5 Resnet cell operations. The neck network was the feature fusion layer that used a three-layer spatial pyramid to fuse information with a large receptive field to a network with a small receptive field. In particular, the 40x40x256 input was transformed into 20x20x512 and 10x10x1024 using two Resnet unit operations, then 10x10x1024 was up-sampled to 20x20x256 using two layers of networks, and 20x20x512 features were stitched into 20x20x768 using another layer of networks. Similarly, the 40x40x256 was also fused to the 20x20x256 features into 40x40x128. The detection head used the three spatial pyramid features exported by the neck network to output the final target location.

We carried out data enrichment to make the data more meaningful. Unlike normal image detection, gastroscopy images do not have an inherent concept of top. **Figure 1** shows the architecture and workflow of the EGC-YOLO system.

A barrier indication is the Threshold-value. When we enter a specified threshold-value into the EGC-YOLO code, EGC-YOLO will display potential areas with scores more than the threshold in red on the screen to alert researchers that this region is suspected of having early gastric cancer lesions. The findings are obtained by comparing it to the green box specified by the manual box.

## Results

### Construction of the Artificial Intelligence Platform System and Image Processing

**Figure 2:**
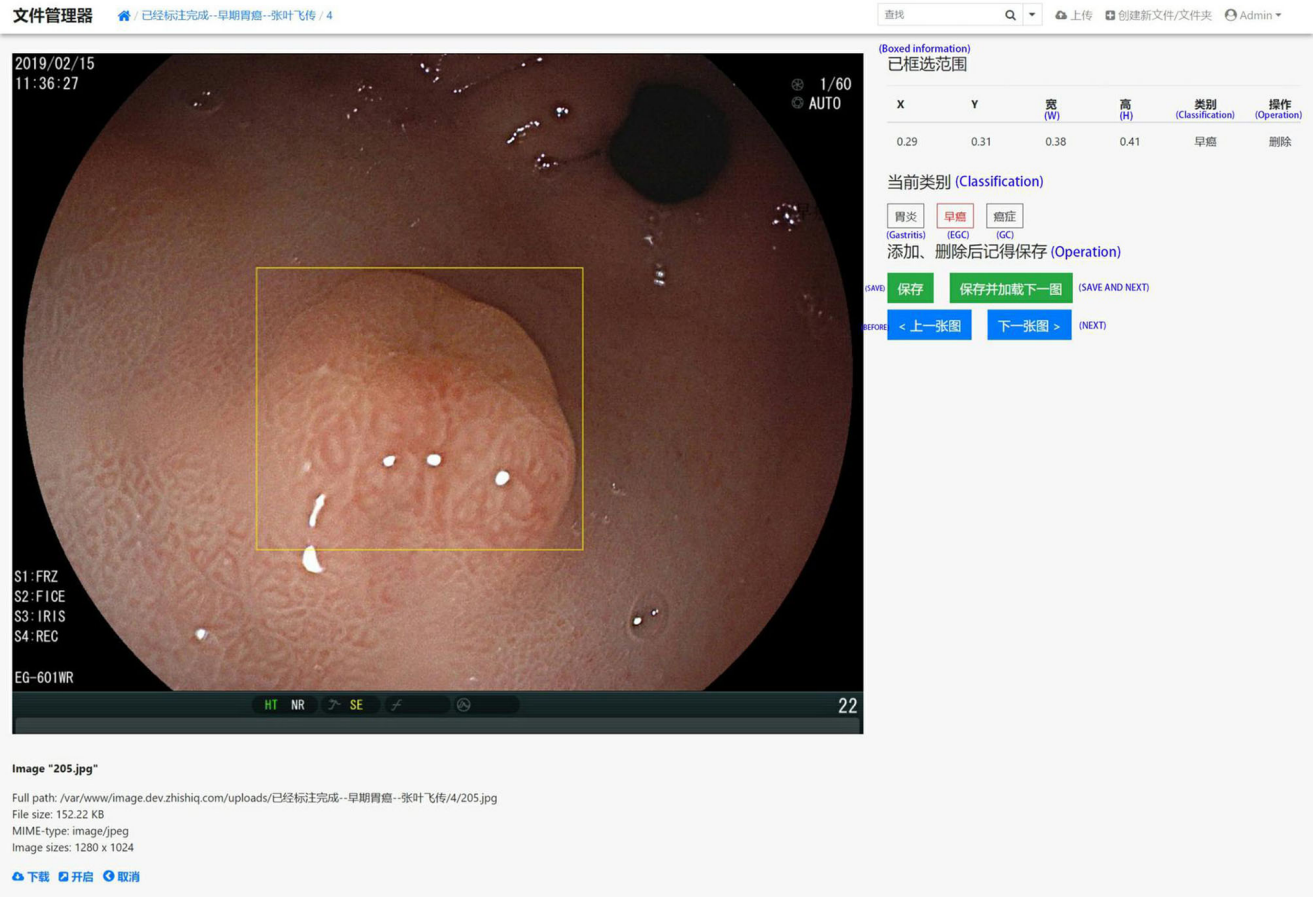
A screenshot of the website database platform and online image annotation we created in this study. The yellow rectangular box shows the diagnosed early gastric cancer lesion. The size and location (x, y, w, and h) of the selected site were displayed in real time on the right side of the image, while the type of lesion (gastritis, early gastric cancer, progressive gastric cancer) of the selected site was annotated. The original information including source, size, format, and resolution is displayed below the image.

**Figure 3:**
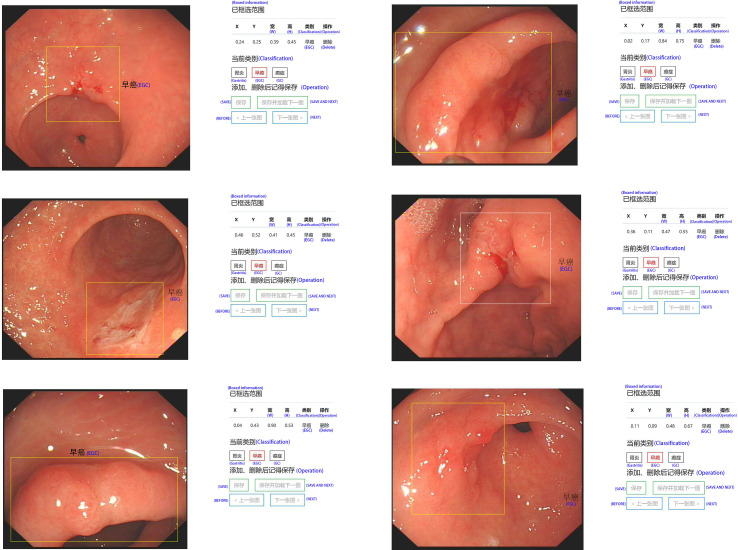
Online labeling of early gastric cancer lesions at 6 different sites.

In order to effectively manage all training and test set data and standardize all images, we created our own website and online image processing tools. We used the tools to label and record the coordinates of early gastric cancer sites for the uploaded images to facilitate subsequent AI training. The platform webpage is shown in **Figures 2** and **3**.

The number of all cases included in the study and the number of valid training images for the three hospitals are presented in **Table 1**.

**Table 1:** Number of cases and images contributed by the three hospitals.

Hospital	YXPH	TSAH	CAHOSH
Non-gastric cancer cases	1431	47	200
Non-gastric cancer images	42200	937	3457
Cases of early gastric cancer	222	48	47
Labeled early gastric cancer images	945	280	159

Yixing People’s Hospital (YXPH), the Second Affiliated Hospital of Soochow University (TSAH), Civil Aviation Hospital of Shanghai (CAHOSH).

### Test Set 1 Results

The test took a total of 7.63 seconds to obtain the specific values of TP, FP, TN, and FN under the corresponding Threshold-values. The Threshold-values were set from 0.01 to 0.99. The results of the first ten rows are shown in **Table 2**, in descending order of the Youden index. When the Threshold-value was 0.02, the maximum Youden index was 0.697727, the accuracy was 0.851541, the sensitivity was 0.853571, the specificity was 0.844156, and the positive predictive value was 0.952191.

**Table 2:** The results of test set 1 showing the top 10 data in descending order of Youden index, with a Threshold-value range of 0.01 to 0.99, containing 99 values.

Threshold	TP	FP	TN	FN	Accuracy	Specificity	Sensitivity	Youden Index	PPV
0.02	239	12	65	41	0.851541	0.844156	0.853571	0.697727	0.952191
0.04	218	7	70	62	0.806723	0.909091	0.778571	0.687662	0.968889
0.03	228	10	67	52	0.826331	0.870130	0.814286	0.684416	0.957983
0.05	210	7	70	70	0.784314	0.909091	0.750000	0.659091	0.967742
0.06	204	7	70	76	0.767507	0.909091	0.728571	0.637662	0.966825
0.08	198	6	71	82	0.753501	0.922078	0.707143	0.629221	0.970588
0.10	194	5	72	86	0.745098	0.935065	0.692857	0.627922	0.974874
0.07	201	7	70	79	0.759104	0.909091	0.717857	0.626948	0.966346
0.09	196	6	71	84	0.747899	0.922078	0.700000	0.622078	0.970297
0.11	191	5	72	89	0.736695	0.935065	0.682143	0.617208	0.974490

### Test Set 2 Results

The test took a total of 6.91seconds to obtain the specific values of TP, FP, TN, and FN under the corresponding Threshold-values. The Threshold-values for this test were taken as 99 values from 0.01 to 0.99. The results are listed in descending order of the Youden index, and the first ten rows of the results are shown in **Table 3**. When the Threshold-value was 0.16 and 0.17, the maximum Youden index was 0.752267, the accuracy was 0.860169, the sensitivity was 0.830189, the specificity was 0.922078, and the positive predictive value was 0.956522.

**Table 3:** The results of Test Set 2 showing the top 10 data in descending order of the Youden Index, with a Threshold-value range of 0.01 to 0.99 and a total of 99 values.

Threshold	TP	FP	TN	FN	Accuracy	Specificity	Sensitivity	Youden Index	PPV
0.16	132	6	71	27	0.860169	0.922078	0.830189	0.752267	0.956522
0.17	132	6	71	27	0.860169	0.922078	0.830189	0.752267	0.956522
0.19	129	5	72	30	0.851695	0.935065	0.811321	0.746386	0.962687
0.18	131	6	71	28	0.855932	0.922078	0.823899	0.745977	0.956204
0.11	139	10	67	20	0.872881	0.870130	0.874214	0.744344	0.932886
0.23	126	4	73	33	0.843220	0.948052	0.792453	0.740505	0.969231
0.24	126	4	73	33	0.843220	0.948052	0.792453	0.740505	0.969231
0.20	128	5	72	31	0.847458	0.935065	0.805031	0.740096	0.962406
0.12	138	10	67	21	0.868644	0.870130	0.867925	0.738055	0.932432
0.10	141	12	65	18	0.872881	0.844156	0.886792	0.730948	0.921569
0.30	122	3	74	37	0.830508	0.961039	0.767296	0.728335	0.976000

**Figure 4:**
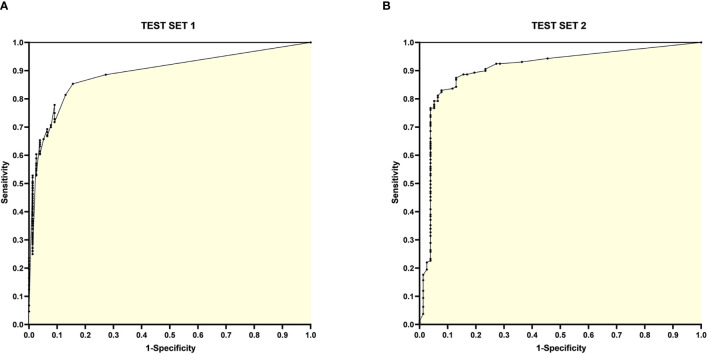
ROC curves for Test Set 1 and 2. **(A)** ROC curve for Test Set 1, with the horizontal coordinate 1-specificity and the vertical coordinate sensitivity, the AUC is 0.8925; **(B)** ROC curve for Test Set 2, with the horizontal coordinate 1-specificity and the vertical coordinate sensitivity, the AUC is 0.9078.

The ROC curves for Test Set 1 and 2 with sensitivity and 1-specificity are shown in **Figure 4**.

### Threshold-Value

**Figure 5:**
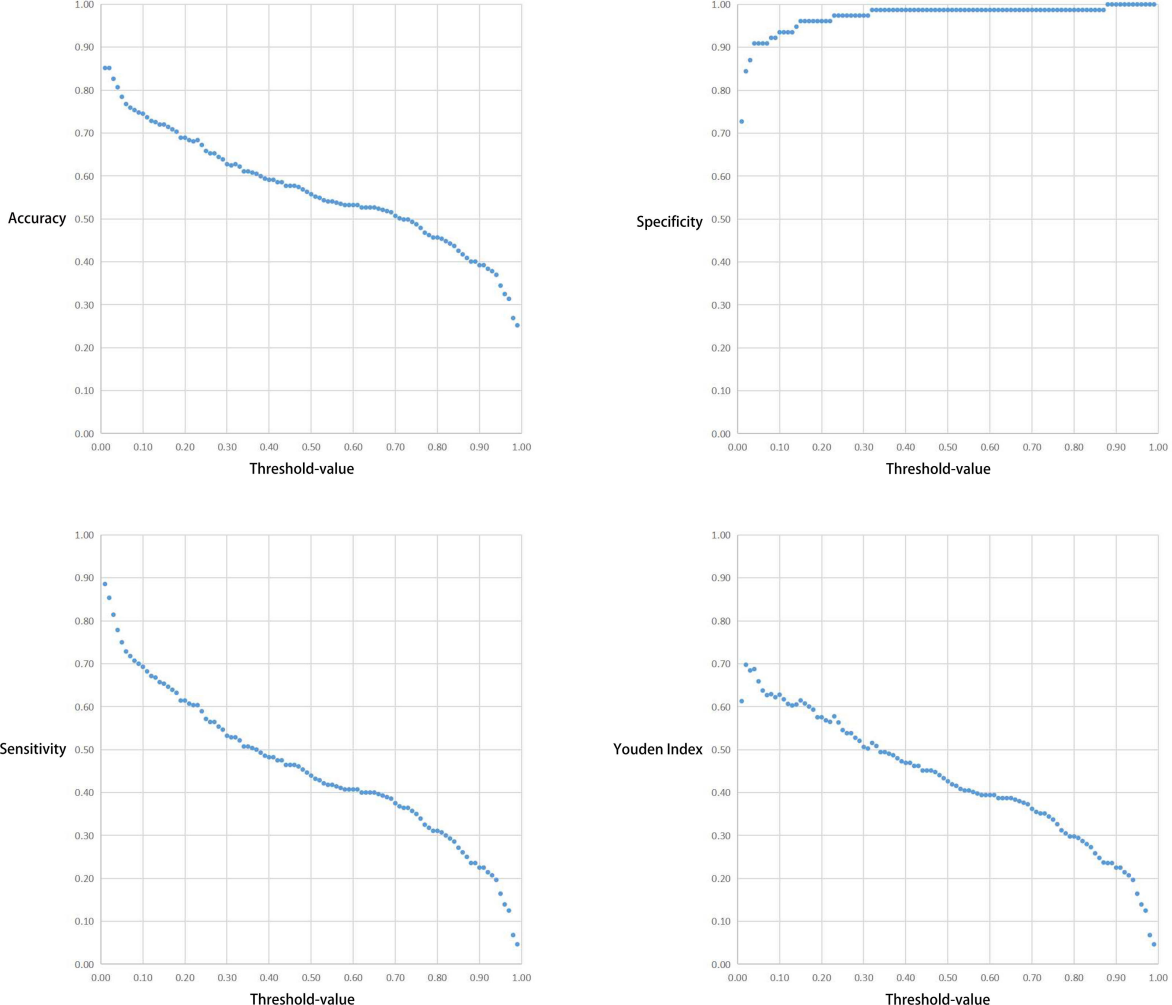
Respective curves of Threshold-values versus Accuracy, Sensitivity, Specificity and Youden Index in Test Set 1.

**Figure 6:**
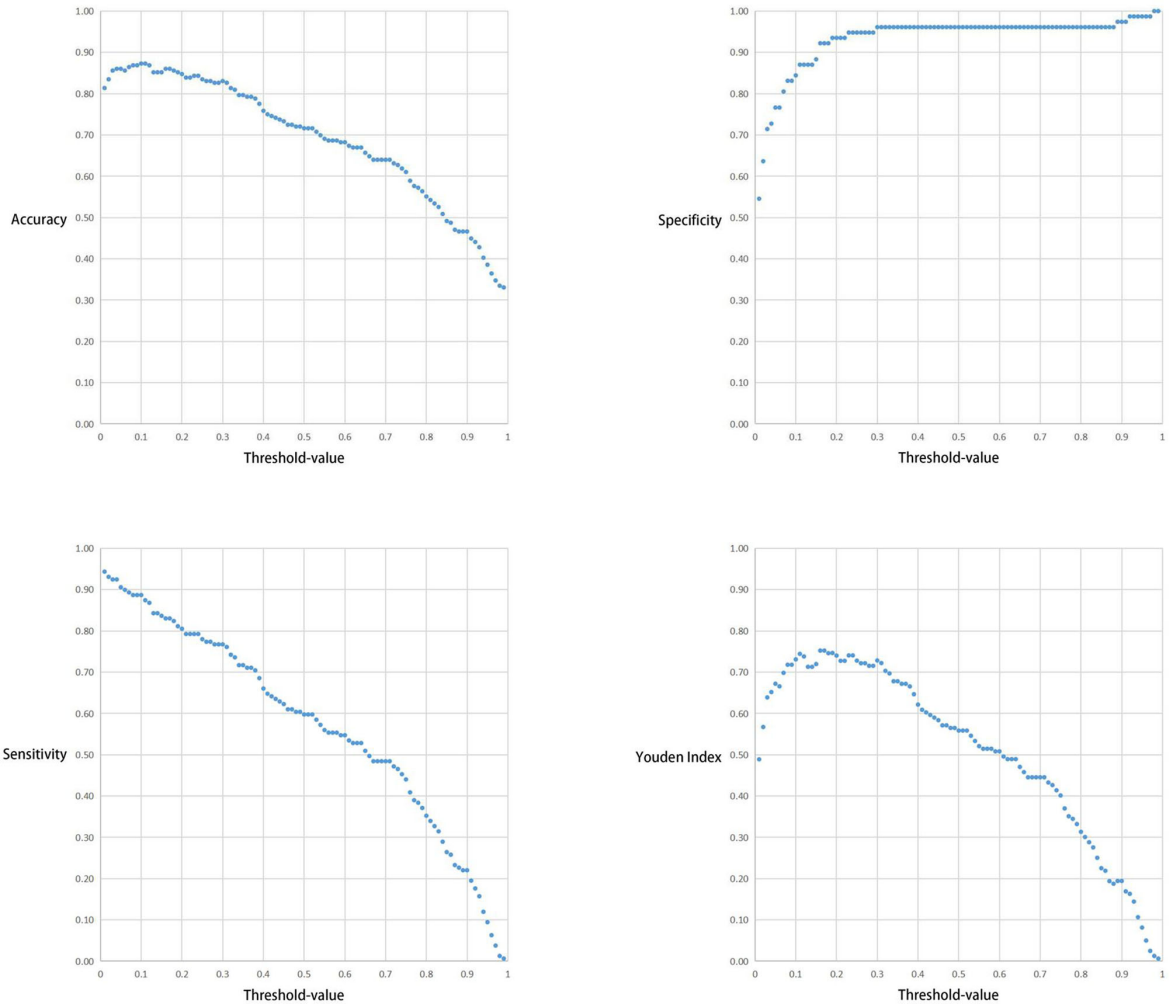
Respective curves of Threshold-values versus Accuracy, Sensitivity, Specificity and Youden Index in Test Set 2.

The correlation between the Threshold-value and sensitivity, specificity, accuracy and Youden index are presented in **Figures 5** and **6**. As the Threshold-value increased, there was a tendency for the accuracy, sensitivity and Youden index values to decrease significantly, while the specificity fluctuated between 0.8 and 1.0 in Test Set 1 and Test Set 2.

Our findings indicate that the YOLOv3 algorithm trained on white light gastroscopy images can identify lesions and frame suspicious lesions with high sensitivity and specificity. In addition, the algorithm can simultaneously identify and frame multiple suspicious lesions in a single image. This is significant because multiple suspicious lesions in one gastroscopic image are most commonly encountered in the clinic. EGC-YOLO provides a more detailed zoning of each gastroscopy image, and each image has several potential boxed areas. When different Threshold-values are selected, the pre-selected boxes with scores greater than the Threshold-value are displayed.

## Discussion

China has a large population that is increasingly choosing gastroscopy as an annual medical checkup routine due to increase in health awareness and the popularization of science. This has helped in the early detection of gastric cancer, but has also greatly increased the workload for endoscopists. The training of qualified endoscopists takes a long time, making it even more difficult for a junior endoscopist to grow into an endoscopist who can independently identify various types of gastric lesions. This leads to the low rates of early gastric cancer diagnosis observed in China. Therefore, there is need to introduce powerful and efficient medical assistance systems to help cope with the extremely heavy endoscopic workload, while improving diagnostic efficiency and avoiding misdiagnosis. Technologies such as artificial intelligence and CNN can cater for this need and AI can be used in the field of capsule endoscopy. The number of images extracted from the video taken by capsule endoscopy is huge, with each patient capable of producing 40,000 to 60,000 images. The analysis of these images by professionals can be time-consuming. However, the use of AI allows images of normal tissues to be automatically filtered out, leaving images of suspicious lesions for further analysis. This greatly improves the efficiency of analyzing the images while reducing the workload of endoscopists.

Several studies have demonstrated the effectiveness and great potential of artificial intelligence in image recognition during gastrointestinal endoscopy. For instance, Ken et al. (28) were able to distinguish between gastric ulcers and gastric cancer by training and testing more than 10,000 images of gastric cancer and gastric ulcers. They used the SSD algorithm with a 16-layer neural network, resulting in a sensitivity of 99% and a specificity of 93.3%. On the other hand, a team led by Gregor (29) was able to detect colon polyps by training 8641 labeled intestinal polyp images using algorithms such as ResNet-50. They then tested 20 colonoscopy videos, resulting in an accuracy of 96.4% and an area under the ROC curve of 0.991. Another team led by Takumi Itoh (30) were able to distinguish HP infections by using the GoogLeNet algorithm to train and test 179 gastroscopy images, resulting in a sensitivity of 86.7% and a specificity of 86.7%. Hiroya Ueyama et al. (31) also investigated the efficacy of artificial intelligence relying on endoscopic NBI images to discriminate between gastritis and gastric cancer. In the study, they trained and tested 2300 images using the ResNet-50 algorithm on over 5400 gastroscopic NBI magnified images, resulting in 98.7% accuracy, 98% sensitivity, and 100% specificity.

The use of convolutional neural network to screen for early gastric cancer can effectively reduce the incidences of misdiagnosis as well as false-positives at the primary screening stage of white light gastroscopy. Further, endoscopic precision investigations such as EUS, magnification endoscopy, and NBI with AI can be performed to clarify the pathology of the lesion.Some investigators have combined AI with magnification endoscopy and NBI and achieved satisfactory results. However, its actual clinical value needs to be investigated further. This is because white light gastroscopy is the main method used in daily endoscopic screening. Further tests such as magnified endoscopy, NBI, EUS, and biopsy are only performed when the lesion is suspected to be gastric cancer. At this stage, misdiagnosis of gastric cancer is rare and therefore the use of CNN to identify gastric cancer lesions according to the above process does not seem to significantly improve the efficiency of gastric cancer detection. In addition, some researchers employed high-quality images as training and test sets (32–34) and obtained satisfactory results. However, the availability of high-quality images in the actual clinical setting is not common in each time, which poses a challenge for AI.

We believe that the selection of images for the training set should be relaxed, and the data access criteria should be refined, so that the images obtained in routine examinations in the clinic can be classified and aggregated before performing AI inspection. This makes AI well adapted for accurate diagnosis of routine endoscopic images. In addition, one gastroscopic image may have multiple suspicious lesions, as is common in the actual clinical setting. The lack of standardized endoscopic equipment and video acquisition devices in local hospitals in China poses a challenge for the use of AI in endoscopy. HD capture cards are not popular, and the quality of gastroscopic imaging in some hospitals is so low that not all images can be obtained at 1080p or even 4k. To overcome these challenges, we used a variety of images of different resolutions in our training set to simulate the nonstandardize quality of images encountered in the routine clinic.

In this study we observed a downward trend in the accuracy, sensitivity and Youden index values as the Threshold-value increased from 0.01 to 0.99. However, the correlation between the Threshold-value and specificity remained stable between 0.8-1.0. The optimal cut-off values for the two test sets were 0.02, 0.16 and 0.17, respectively. This suggested that Threshold-value need to be adjusted before detecting image data from different databases, to obtain the most accurate test results. We do not agree with the subjective Threshold-value set as 0.5 in some studies as it produced poor results in our study. When we set the Threshold-value as 0.5, the accuracy, specificity and sensitivity of Test Set 1 and Test Set 2 were 0.557423 and 0.716102, 0.987013 and 0.961039 and 0.439286 and 0.597484, respectively, indicating poor test performance. Therefore, in cases where the specificity cannot be significantly improved, a small Threshold-value is associated with high EGC-YOLO test efficiency. For such a result, we found through careful analysis that the threshold-value was very different from the traditional human biochemical indicators, for example, AFP≥400μg/L in the diagnosis of primary liver cancer and blood amylase > 300U/L in the diagnosis of acute pancreatitis. Because the gastroscopic images of the test set came from different hospitals, they were taken by different equipment. Their baseline values, such as resolution, color saturation and brightness, are different. For the AI system, the nature of data from different data sets is different, so it is necessary to adjust the best threshold value to give full play to the best performance of EGC-YOLO. Images from different data sources need to have their own specific threshold-value during detection. We found that it was not rigorous behavior to set a threshold according to human experience in previous experiments.

There is a huge potential for the application of artificial intelligence in the field of gastrointestinal endoscopy, but many studies are still in the primary trial stage and few are actually put into clinical application. In China, artificial intelligence has been developing rapidly in recent years, and its application in the medical field is likely to increase in the next 5-10 years. Therefore, the prospect of intelligent medical care in China is bright. The value of artificial intelligence lies in its ability to take over the repetitive nature of a single human task, rather than completely replacing human intervention. The advantages of artificial intelligence (i.e. fast and efficient, fatigue-free, omission-free handling of single daily tasks) will free doctors to do more creative medical research.

This experiment is a retrospective study, and the relevant conclusion is to manually screen the image data retained by the endoscopic center of the hospital for many years and compare it with the pathological standard, based on these results, we will add prospective study to verify the reliability and clinical application value of this study in the future.

## Data Availability Statement

The original contributions presented in the study are included in the article/supplementary material. Further inquiries can be directed to the corresponding author.

## Ethics Statement

This project was reviewed and approved by the Medical Ethics Committee of Yixing People's Hospital. This study followed the standards of the Medical Ethics Committee and signed the medical ethics agreement. All images were anonymised before inclusion to protect the privacy of the patients. Informed consent was not required from patients whose images were retrospectively obtained from the image databases at each hospital involved in this study.

## Author Contributions

Conceptualization, ZY and BL. Methodology, ZY. Software, BL. Formal analysis, ZY and BL. Investigation, TJ, YZ, and SL. Resources, BM. Data curation, BL. Writing—original draft preparation, ZY. Writing—review and editing, WC. Visualization, ZY and BL. Supervision, WC and BM. Project administration, WC. Funding acquisition, ZY. All authors have read and agreed to the published version of the manuscript.

## Funding

This research was funded by the Foundation of Clinical Science and Technology of Wuxi, No.Q202062.

## Conflict of Interest

Author BL is employed by Microsoft Ltd.

The remaining authors declare that the research was conducted in the absence of any commercial or financial relationships that could be construed as a potential conflict of interest.

## Publisher’s Note

All claims expressed in this article are solely those of the authors and do not necessarily represent those of their affiliated organizations, or those of the publisher, the editors and the reviewers. Any product that may be evaluated in this article, or claim that may be made by its manufacturer, is not guaranteed or endorsed by the publisher.
